# Fifteen-year changes in health-related quality of life after bariatric surgery and non-surgical obesity treatment

**DOI:** 10.1038/s41366-024-01572-w

**Published:** 2024-06-20

**Authors:** Hanna Konttinen, Kajsa Sjöholm, Lena M. S. Carlsson, Markku Peltonen, Per-Arne Svensson

**Affiliations:** 1 Social Psychology, Faculty of Social Sciences, University of Helsinki, Helsinki, Finland; 2 Institute of Medicine, Sahlgrenska Academy at University of Gothenburg, Gothenburg, Sweden; 3 Finnish Institute for Health and Welfare, Helsinki, Finland; 4 Institute of Health and Care Sciences, Sahlgrenska Academy at University of Gothenburg, Gothenburg, Sweden

**Keywords:** Bariatric surgery, Weight management

## Abstract

**Background:**

Evidence on the long-term (≥10 years) development of health-related quality of life (HRQoL) following bariatric surgery is still limited and mainly based on small-scale studies. This study aimed to investigate (1) 15-year changes in mental, physical, social, and obesity-related HRQoL after bariatric surgery and non-surgical obesity treatment; and (2) whether sociodemographic factors and pre-operative health status are associated with 15-year HRQoL changes in the surgery group.

**Methods:**

Participants were from the non-randomized, prospective, controlled Swedish Obese Subjects study. The surgery group (*N* = 2007, per-protocol) underwent gastric bypass, banding or vertical banded gastroplasty, and matched controls (*N* = 2040) received usual obesity care. Recruitment took place in 1987–2001 and measurements (including six HRQoL scales) were administered before treatment and after 0.5, 1, 2, 3, 4, 6, 8, 10 and 15 years. Multilevel mixed-effect regression models using all observations for estimation were conducted.

**Results:**

Surgical patients experienced greater 15-year improvements in perceived health and overall mood, and greater reductions in depression, obesity-related problems, and social interaction limitations than controls (all *p* < 0.001, adjusted for baseline differences). Effect size (ES) was classified as large only for obesity-related problems (ES = 0.82). At the 15-year follow-up, surgical patients reported better perceived health (*p* < 0.001) and less obesity-related problems (*p* = 0.020) than controls. In the surgery group, patients with baseline diabetes had smaller 15-year reductions in social interaction limitations (*p* < 0.001) and depression (*p* = 0.049) compared to those without baseline diabetes. Although surgical patients with a history of psychiatric disorder reported lower HRQoL than those without such history over the 15-year follow-up, there were no significant differences in the long-term improvements between the two groups (*p* = 0.211–0.902).

**Conclusions:**

Over 15 years, surgical patients experienced more positive development of HRQoL compared to those receiving usual care. This difference was large for obesity-related problems, but otherwise the differences were small. Patients with pre-operative diabetes might be at increased risk for smaller long-term HRQoL improvements.

## Introduction

Health-related quality of life (HRQoL) is often impaired in individuals with obesity who seek or undergo bariatric surgery [1, 2]. Since HRQoL is a multidimensional construct encompassing perceived health in physical, mental and social domains of functioning, several factors are likely to contribute to impaired HRQoL in candidates for bariatric surgery. Individuals with severe obesity frequently encounter weight-based discrimination and suffer from co-morbidities (e.g., type 2 diabetes, cardiovascular diseases) which can negatively affect HRQoL [3, 4]. Research further implies that a desire for improved quality of life and psychosocial functioning are important reasons to seek bariatric surgery in a significant portion of patients [5, 6]. Studying long-term HRQoL outcomes after surgery is therefore highly relevant.

While it is well demonstrated that bariatric surgery leads to substantial, long-term weight loss and improvements in obesity-related co-morbidities [7–10], knowledge on the long-term post-operative HRQoL development is still limited and mainly based on studies of smaller scale. Andersen et al. identified 7 studies with sample sizes ranging from 44 to 655 patients in their systematic review of prospective studies reporting HRQoL data at least 5 years after bariatric surgery [11]. The findings suggested that HRQoL typically improves during the first and second post-operative years, followed by a gradual decline that appears to stabilize 5 years post-operatively [11]. Another systematic review and meta-analysis on the long-term (≥5 years) HRQoL outcomes reported fairly similar results [12]. However, long-term HRQoL changes may also vary between different domains of functioning. In the study by Kolotkin et al. [13] gastric bypass patients showed very large and significant enhancements in weight-related and physical HRQoL at 12 years relative to baseline, while improvements in mental or psychosocial HRQoL were small. Moreover, a higher risk of alcohol and substance abuse [14, 15], an increase in psychiatric illness presentations [16], and a higher risk of self-harm and suicide events [17, 18] have been observed after bariatric surgery indicating that some patients may experience a deteriorated HRQoL post-operatively.

It may be speculated that certain patient characteristics are associated with poorer long-term HRQoL outcomes. The evidence is currently scarce but is required to better identify those patients who could benefit from more intensive post-operative psychosocial care or should be advised not to undergo bariatric surgery. A recent study conducted in 7000 gastric bypass patients from the Scandinavian Obesity Surgery Register found that higher age and greater weight loss were related to larger 5-year improvement in HRQoL, whereas pre-operative medication for depression and suffering a post-operative complication were associated with less improvement in HRQoL [19]. Moreover, previous results from a smaller subset of participants from the Swedish Obese Subjects (SOS) study showed that 10-year changes in HRQoL after surgery largely followed phases of weight loss, weight regain and weight stability, although other potential predictors of HRQoL development were not explored [20].

The aim of the present analyses was to update and extend previous findings from the SOS study [20]. More precisely, we aimed (1) to examine 15-year changes in mental, physical, social and obesity-related HRQoL after bariatric surgery and non-surgical obesity treatment; and (2) to identify baseline sociodemographic and health-related factors that are associated with 15-year HRQoL changes in the bariatric surgery group. Since larger weight loss, but also increased risk of side effects have been observed after gastric bypass [10, 15, 17, 18, 21], we additionally examined the effect of surgery type (banding, vertical banded gastroplasty, gastric bypass) on the long-term HRQoL development.

## Methods

### Participants and study design

Participants were from the SOS study. It is a matched, non-randomized, prospective intervention trial comparing bariatric surgery with usual care for patients with obesity [7, 10]. Between September 1, 1987 and January 31, 2001, altogether 4047 patients with obesity were enrolled at 25 surgical departments and 480 primary health care centers in Sweden. Patients were recruited from a matching examination completed by 6905 patients of which 5335 met the inclusion criteria. The inclusion and exclusion criteria were identical for the surgery and controls groups. The inclusion criteria were age 37–60 years and BMI ≥ 34 kg/m^2^ for men and BMI ≥ 38 kg/m^2^ for women at recruitment. The exclusion criteria comprised earlier surgery for gastric or duodenal ulcer, earlier bariatric surgery, gastric ulcer or myocardial infarction during the past 6 months, ongoing malignancy, active malignancy during the past 5 years, bulimic eating pattern, drug or alcohol abuse, psychiatric or cooperative problems contraindicating bariatric surgery and other contraindicating conditions (e.g., chronic glucocorticoid or anti-inflammatory treatment). The surgery group consisted of 2010 individuals electing the surgery as a co-decision with the physician (*N* = 2007 per protocol as 3 patients were never operated). The matched control group of 2037 individuals (*N* = 2040 per protocol) was created using 18 matching variables [10]. The matching algorithm utilized the method of sequential treatment assignment and selected controls in a way that the current mean values of the matching variables in the control group became as similar as possible to the current mean values in the surgery group [22]. Thus, although the surgical patient and their matched control patient always started the study on the day of surgery, the matching was not performed at an individual level. The primary outcome in the SOS study was all-cause mortality, and the study had 80% power (*p* = 0.05) to detect a 23% difference in mortality between 2000 surgical patients and 2000 controls followed up for 10 years [23].

Surgery type was determined by the surgeon and included nonadjustable or adjustable banding (*N* = 376), vertical banded gastroplasty (*N* = 1365), and gastric bypass (*N* = 266). Control patients were offered usual obesity care at their regular primary health care center. The treatment was not standardized and varied according to the local practices from sophisticated lifestyle intervention and behavior modification to a lack of specific treatment and health monitoring only. All patients were invited to attend a health examination and complete various questionnaires prior to treatment and after 0.5, 1, 2, 3, 4, 6, 8, 10 and 15 years.

The SOS study was registered at clinicaltrials.gov as NCT01479452. Seven Swedish regional ethics review boards (Gothenburg, Karolinska Institute, Linköping, Lund, Umeå, Uppsala, Örebro) approved the study protocol. Written or oral informed consent was obtained from all patients. All the procedures performed were in accordance with the standards of the ethical committee and with the 1964 Helsinki declaration [24].

### Measures

#### Sociodemographic factors

Participants self-reported their level of education and relationship status at baseline. Education level (basic vs. upper secondary/university education) and relationship status (non-married/no partner vs. married/partner) were both dichotomized for the main analyses. Sex (men vs. women) and age (<47.8-year-olds vs. ≥47.8-year-olds based on the sample median age) were also treated as dichotomous variables.

#### Pre-operative health status

Diabetes was defined as a fasting blood glucose level of at least 6.1 mmol per litre (110 mg per deciliter) or self-reported use of a prescribed antidiabetic medication at baseline. Patients who had health care visits for mental disorders, substance abuse or non-fatal self-harm prior to participation in the SOS study and/or had used psychiatric drugs during the last 3 months were defined as having a history of psychiatric disorder. These health care visits were identified from the National Patient Register using International Classification of Diseases (ICD) codes (Supplementary Table 1). Psychiatric drug use was assessed via self-report at baseline and the responses were classified according to the Anatomical Therapeutic Chemical (ATC) classification system codes (N05: psycholeptics, N06: psychoanaleptics).

HRQoL was assessed using the same validated self-administered scales as in the study by Karlsson et al. [20]. These six scales, detailed below, have been administered at each study visit.

#### Physical HRQoL

Perceived health status was measured by the 9-item Current Health Scale of the General Health Rating Index [25]. The original five-point response format was modified in the SOS study to a four-point scale with two acceptance and two rejection alternatives. The nine items (e.g., “My health is excellent”) were summed up into a total score ranging between 0 and 100. Higher scores indicate better perceived health status.

#### Mental HRQoL

Overall mood was measured by the short version of the Mood Adjective Check List (MACL) [26]. MACL contains 38 adjectives on a four-point response scale with two acceptance and two rejection alternatives. It measures three major bipolar dimensions of mood that are highly correlated: pleasantness/unpleasantness, activation/deactivation, and calmness/tension. A total score of all items was calculated to describe overall mood. Scores range between 1 and 4 with higher scores indicating more positive mood states. Depression and anxiety were assessed using the Hospital Anxiety and Depression scale (HAD) [27]. HAD is a screening tool for anxiety and depression disorders in somatically ill patients, which contains 14 items on a four-point response scale. The items were summed up into separate anxiety and depression scores ranging between 0 and 21. Higher scores represent more symptoms.

#### Social and obesity-related HRQoL

The Social Interaction category (SI) of the Sickness Impact Profile (SIP) was used to assess health-related limitations in social life [28]. SI contains 20 statements (e.g., “I am doing fewer social activities with groups of people”) on quality and quantity of social interaction within the family, among friends and in the community. Scores represent the weighted sum of endorsed items and range between 0 and 100. Higher scores indicate more limitations in social interaction. The impact of obesity on psychosocial functioning was measured by the 8-item Obesity-related Problems scale (OP) [29]. Respondents are asked to indicate on a four-point scale how bothered they are by their obesity in a broad range of social activities (e.g., private gatherings at home, going to restaurants, participation in community activities, holidays away from home, trying on and buying clothes, bathing in public places, intimate relations). Responses were aggregated to a total score that ranges from 0 (no impairment) to 100 (maximum impairment).

### Statistical methods

Mean changes in each HRQoL scale during 15 years of follow-up were analyzed with multilevel mixed-effect regression models using all observations for estimation. Treatment group × time interactions were calculated to test differences between the surgery group and the control group in these HRQoL changes (adjusted for baseline differences in each scale). To examine the effects of sociodemographic factors (age, sex, relationship status, education level), pre-operative health status (type 2 diabetes, history of psychiatric disorder) and surgery type (banding as a reference category) on HRQoL changes in the surgery group, interactions between each baseline predictor and time were tested. These analyses were conducted separately for each HRQoL scale and adjusted for sex, age, baseline BMI and the respective HRQoL scale at baseline. Assumptions of the models were evaluated by analysis of residuals. The threshold for statistical significance was set at *p* < 0.05 (two-sided). No correction for multiple testing was done. All analyses were prespecified, and we report the results of them all here.

Effect sizes (ES) of within-group 15-year changes were calculated as in the study by Karlsson et al. [20]: a mean change between the assessments was divided by the standard deviation of change. ES of between-group differences were computed as a difference in 15-year mean change between the control and surgery groups divided by the pooled standard deviation of change. ES was judged against the following criteria: trivial (0 to <0.2), small (0.2 to <0.5), moderate (0.5 to <0.8) and large (≥0.8) [30].

## Results

Baseline descriptive characteristics of the surgery and control groups are shown in Table 1. Surgical patients had lower mean age (47.2 vs. 48.7, *p* < 0.001), less often university education (13% vs. 21%, *p* < 0.001), higher mean BMI (42.4 vs. 40.1, *p* < 0.001), and more often diabetes (17% vs. 13%, *p* < 0.001) and history of psychiatric disorder (19% vs. 16%, *p* = 0.038) compared to control patients. On average, BMI was reduced by approximately 15% from baseline to 15 years in surgical patients, while there was no major change in control patients (Supplementary Fig. 1). Compared to control patients, surgical patients reported lower HRQoL at baseline in terms of all six scales (all *p* < 0.001) (Table 2).

**Table 1:** Baseline descriptive characteristics by the treatment group in the Swedish Obese Subjects study.

Variable	Control (*N* = 2040)		Surgery (*N* = 2007)^a^		*p* value^b^
	Mean/%	SD/*N*	Mean/%	SD/*N*	
Age (years)	48.7	6.3	47.2	5.9	<0.001
Men (%)	29.1	593	29.2	587	0.917
Married or partner (%)	75.3	1527	72.9	1460	0.098
Education					<0.001
Basic (%)	59.9	1222	69.4	1393	
Upper secondary (%)	19.0	387	17.8	357	
University (%)	21.1	431	12.8	257	
Diabetes (%)	12.9	263	17.2	344	<0.001
BMI (kg/m^2^)	40.1	4.7	42.4	4.5	<0.001
History of psychiatric disorder (%)	16.2	331	18.7	376	0.038

^a^Type of surgery: nonadjustable or adjustable banding (376 patients), vertical banded gastroplasty (1365 patients), and gastric bypass (266 patients).

^b^Differences between the treatment groups were tested using *t*-tests (continuous variables) or Fisher’s exact tests (categorical variables).

**Table 2:** Mean levels and changes in HRQoL scales over 15 years of follow-up in the SOS control and surgery groups estimated using mixed-models.

	Control (*N* = 2040)		Surgery (N = 2007)		*p* value	Between-group difference in 15-yr change	
	Mean	95% CI	Mean	95% CI		Mean (95% CI)^f^	ES^g,c^
Perceived health^a^							
Baseline	57.5	(56.4–58.7)	50.1	(48.9–51.2)	<0.001^d^		
10-yr follow-up	53.7	(52.3–55.2)	57.2	(55.8–58.5)	0.001^d^		
15-yr follow-up	50.6	(48.7–52.6)	55.6	(53.9–57.4)	<0.001^d^		
15-yr change	−6.9***	(−8.9 to −4.9)	5.6***	(3.8–7.4)	<0.001^e^	12.5 (9.8–15.2)	0.42
ES (within-group)^b,c^	−0.19		0.22				
Anxiety^a^							
Baseline	5.4	(5.2–5.6)	6.0	(5.8–6.2)	<0.001^d^		
10-yr follow-up	4.2	(4.0–4.5)	4.7	(4.5–5.0)	0.001^d^		
15-yr follow-up	4.2	(3.9–4.4)	4.5	(4.3–4.8)	0.072^d^		
15-yr change	−1.2***	(−1.5 to −1.0)	−1.5***	(−1.7 to −1.2)	0.214^e^	−0.2 (−0.6–0.1)	0.10
ES (within-group)	−0.31		−0.39				
Depression^a^							
Baseline	4.1	(4.0–4.3)	5.3	(5.1–5.4)	<0.001^d^		
10-yr follow-up	3.8	(3.6–4.0)	3.8	(3.7–4.0)	0.732^d^		
15-yr follow-up	3.9	(3.6–4.2)	4.1	(3.9–4.4)	0.181^d^		
15-yr change	−0.2*	(−0.5–0.0)	−1.1***	(−1.4 to −0.9)	<0.001^e^	−0.9 (−1.2 to −0.6)	0.30
ES (within-group)	−0.04		−0.30				
Overall mood^a^							
Baseline	3.0	(3.0–3.0)	2.9	(2.8–2.9)	<0.001^d^		
10-yr follow-up	3.1	(3.0–3.1)	3.0	(3.0–3.1)	0.036^d^		
15-yr follow-up	3.1	(3.0–3.1)	3.0	(3.0–3.1)	0.191^d^		
15-yr change	0.1***	(0.0–0.1)	0.2***	(0.1–0.2)	<0.001^e^	0.1 (0.0–0.1)	0.21
ES (within-group)	0.14		0.31				
Obesity-related problems^a^							
Baseline	41.4	(40.2–42.5)	58.9	(57.7–60.0)	<0.001^d^		
10-yr follow-up	32.1	(30.8–33.5)	29.4	(28.1–30.7)	0.005^d^		
15-yr follow-up	32.8	(31.0–34.6)	29.9	(28.2–31.5)	0.020^d^		
15-yr change	−8.6***	(−10.3 to −6.9)	−29.0***	(−30.5 to −27.4)	<0.001^e^	−20.4 (−22.7 to −18.0)	0.82
ES (within-group)	−0.35		−1.02				
Social interaction limitations^a^							
Baseline	9.1	(8.6–9.6)	14.0	(13.5–14.5)	<0.001^d^		
10-yr follow-up	8.3	(7.7–9.0)	8.4	(7.8–9.0)	0.883^d^		
15-yr follow-up	8.8	(8.0–9.6)	9.2	(8.5–10.0)	0.448^d^		
15-yr change	−0.3	(−1.1–0.5)	−4.7***	(−5.5 to −4.0)	<0.001^e^	−4.5 (−5.6 to −3.4)	0.37
ES (within-group)	0.01		−0.33				

***p < 0.001; *p < 0.05 for 15-year change within the treatment group.

^a^Perceived health: higher scores represent better perceived health (score range 0–100). Anxiety and depression: higher scores represent more symptoms (score range 0–21). Overall mood: higher scores represent more positive mood states (score range 1–4). Obesity-related problems and social interaction limitations: higher scores represent more dysfunction (score range 0–100).

^b^Within-group effect size (ES) of 15-year change. Calculated by dividing the 15-year mean change by the standard deviation of change.

^c^Effect size (ES) was judged against the following criteria: trivial (0 to <0.2), small (0.2 to <0.5), moderate (0.5 to <0.8) and large (≥0.8) [30].

^d^Difference in mean level between the treatment groups.

^e^Difference in 15-year change between the treatment groups (adjusted for baseline differences in each HRQoL scale).

^f^Adjusted for baseline differences in each HRQoL scale.

^g^Between-group effect size (ES) of 15-year change. Calculated by dividing the difference in 15-year mean change between the control and surgery groups by the pooled standard deviation of change.

### Changes within the control group

**Fig. 1:**
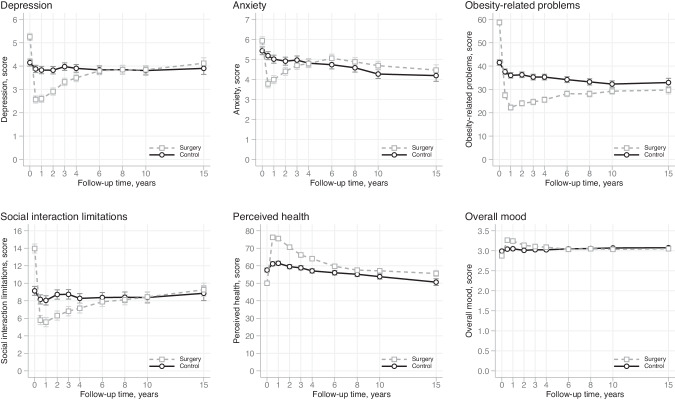
Absolute levels of HRQoL (mean score and 95% CI) by the treatment group over 15 years of follow-up. Higher scores for perceived health (score range 0–100) and overall mood (score range 1–4) represent better HRQoL. Higher scores for anxiety and depression (score range 0–21) and obesity-related problems and social interaction limitations (score range 0–100) represent worse HRQoL.

In the control group, perceived health worsened from baseline to 15 years (*p* < 0.001). In contrast, overall mood (*p* < 0.001) improved, and depression (*p* = 0.049), anxiety (*p* < 0.001) and obesity-related problems (*p* < 0.001) decreased (Table 2 and Fig. 1). ES estimates (within-group) indicated that the changes in obesity-related problems and anxiety were small, while the changes in depression, overall mood and perceived health were trivial (Table 2). The 15-year mean change in social interaction was not significant (*p* = 0.441).

### Changes within the surgery group

In the surgery group, perceived health and overall mood improved, and depression, anxiety, obesity-related problems and social interaction limitations decreased from baseline to 15 years (all *p* < 0.001) (Table 2 and Fig. 1). ES estimates (within-group) indicated that the change in obesity-related problems was large in magnitude (ES = −1.02), and the changes in perceived health, depression, anxiety, overall mood and social interaction were small (Table 2).

### Comparison between the surgery and control groups

Compared to control patients, surgical patients experienced greater improvements in perceived health and overall mood, and greater decreases in depression, obesity-related problems and social interaction limitations (all *p* < 0.001, adjusted for baseline differences in the respective HRQoL scale) over 15 years (Table 2). The between-group differences were small in magnitude, although a larger difference for obesity-related problems (ES = 0.82) was noted. The 15-year mean change in anxiety did not differ significantly (*p* = 0.214) between the treatment groups. At the 15-year follow-up, surgical patients reported better perceived health (*p* < 0.001) and less obesity-related problems (*p* = 0.020) than control patients (Table 2). In contrast, there were no statistically significant differences in anxiety (*p* = 0.072), depression (*p* = 0.181), overall mood (*p* = 0.191) and social interaction (*p* = 0.448) between the groups. Figure 1 reveals key insights into the group comparisons, showing that surgical patients initially had lower HRQoL than control patients. The differences in HRQoL changes were particularly notable in the first and second follow-up years, where surgical patients exhibited greater improvements. Over time, the mean HRQoL levels and change patterns of the two groups became more similar.

### Baseline predictors of changes within the surgery group

Multilevel mixed-effect regression models using baseline factors as predictors and performed only in the surgery group detected a few significant associations (Table 3). Compared to surgical patients without pre-operative diabetes, those with diabetes experienced smaller decreases in social interaction limitations (−5.1 vs. −0.7, *p* for difference in change <0.001) and depression (−1.2 vs. −0.5, *p* for difference in change = 0.049) from baseline to 15 years. Banding resulted in a smaller reduction in obesity-related problems compared to gastric bypass (−27.1 vs. −35.3, *p* for difference in change = 0.009), but not compared to vertical banded gastroplasty (−27.1 vs. −28.2, *p* for difference in change = 0.525) (Supplementary Table 2). Age, sex, education level, relationship status or history of psychiatric disorder were not significantly related to 15-year changes in any of the HRQoL scales (Table 3 and Supplementary Table 2).

**Table 3:** Fifteen-year mean changes in HRQoL scales by baseline age, sex, history of psychiatric disorder, and diabetes status in the SOS surgery group estimated using mixed-models^a^.

	Age		Sex		History of psychiatric disorder		Diabetes	
	<47.8 years	≥47.8 years	Women	Men	No	Yes	No	Yes
	*N* = 1104	*N* = 903	*N* = 1420	*N* = 587	*N* = 1631	*N* = 376	*N* = 1656	*N* = 344
Perceived health^b^								
Mean	3.5	6.5	4.7	5.5	4.5	7.7	4.9	4.4
95% CI	1.0–6.0	3.8–9.3	2.5–6.9	2.1–8.9	2.4–6.5	3.0–12.4	2.9–6.9	−0.3–9.1
*p*^d^	0.113		0.693		0.211		0.848	
Anxiety^c^								
Mean	−1.3	−1.4	−1.2	−1.7	−1.4	−1.3	−1.4	−1.2
95% CI	−1.7 to −1.0	−1.8 to −1.0	−1.5 to −0.9	−2.2 to −1.2	−1.7 to −1.1	−1.9 to −0.6	−1.7 to −1.1	−1.8 to −0.5
*p*^d^	0.830		0.103		0.725		0.455	
Depression^c^								
Mean	−1.1	−1.0	−1.2	−0.7	−1.1	−1.1	−1.2	−0.5
95% CI	−1.4 to −0.8	−1.4 to −0.7	−1.5 to −0.9	−1.2 to −0.3	−1.3 to −0.8	−1.7 to −0.5	−1.4 to −0.9	−1.1–0.1
*p*^d^	0.872		0.092		0.881		0.049	
Overall mood^b^								
Mean	0.2	0.2	0.2	0.2	0.2	0.2	0.2	0.1
95% CI	0.1–0.2	0.1–0.2	0.1–0.2	0.1–0.2	0.1–0.2	0.1–0.3	0.1–0.2	0.0–0.2
*p*^d^	0.436		0.641		0.902		0.126	
Obesity-related problems^c^								
Mean	−29.1	−27.9	−29.1	−27.0	−28.3	−30.4	−28.8	−27.9
95% CI	−31.4 to −26.8	−30.4 to −25.4	−31.1 to −27.1	−30.1 to −23.9	−30.1 to −26.5	−34.7 to −26.1	−30.7 to −27.0	−32.3 to −23.6
*p*^d^	0.479		0.274		0.380		0.702	
Social interaction limitations^c^								
Mean	−5.1	−3.6	−4.6	−3.9	−4.5	−4.2	−5.1	−0.7
95% CI	−6.2 to −4.0	−4.7 to −2.4	−5.6 to −3.7	−5.3 to −2.4	−5.3 to −3.6	−6.2 to −2.2	−6.0 to −4.3	−2.7 to 1.3
*p*^d^	0.063		0.392		0.837		<0.001	

^a^Analyses adjusted for sex, age, baseline BMI and baseline HRQoL scale.

^b^Increased scores represent improved perceived health and overall mood, respectively.

^c^Decreased scores represent reduced anxiety, depression, obesity-related problems and social interaction limitations, respectively.

^d^Difference in 15-year change between the groups (adjusted for sex, age, baseline BMI and the respective HRQoL scale at baseline).

### Absolute levels of HRQoL by subgroups

**Fig. 2:**
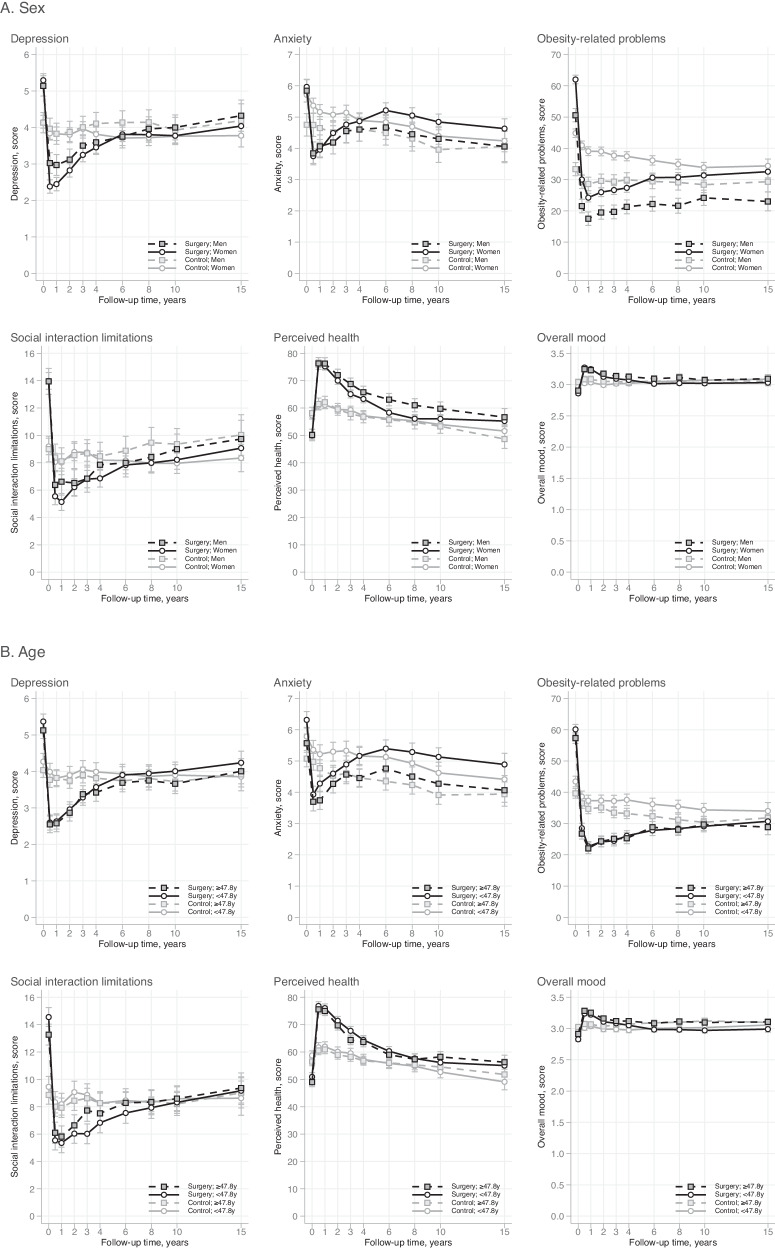
Absolute levels of HRQoL by baseline sociodemographic factors and the treatment group over 15 years of follow-up. **A** Mean scores and 95% CIs by sex in the SOS surgery and control groups; **B** mean scores and 95% CIs by age in the SOS surgery and control groups. Higher scores for perceived health (score range 0–100) and overall mood (score range 1–4) represent better HRQoL. Higher scores for anxiety and depression (score range 0–21) and obesity-related problems and social interaction limitations (score range 0–100) represent worse HRQoL.

**Fig. 3:**
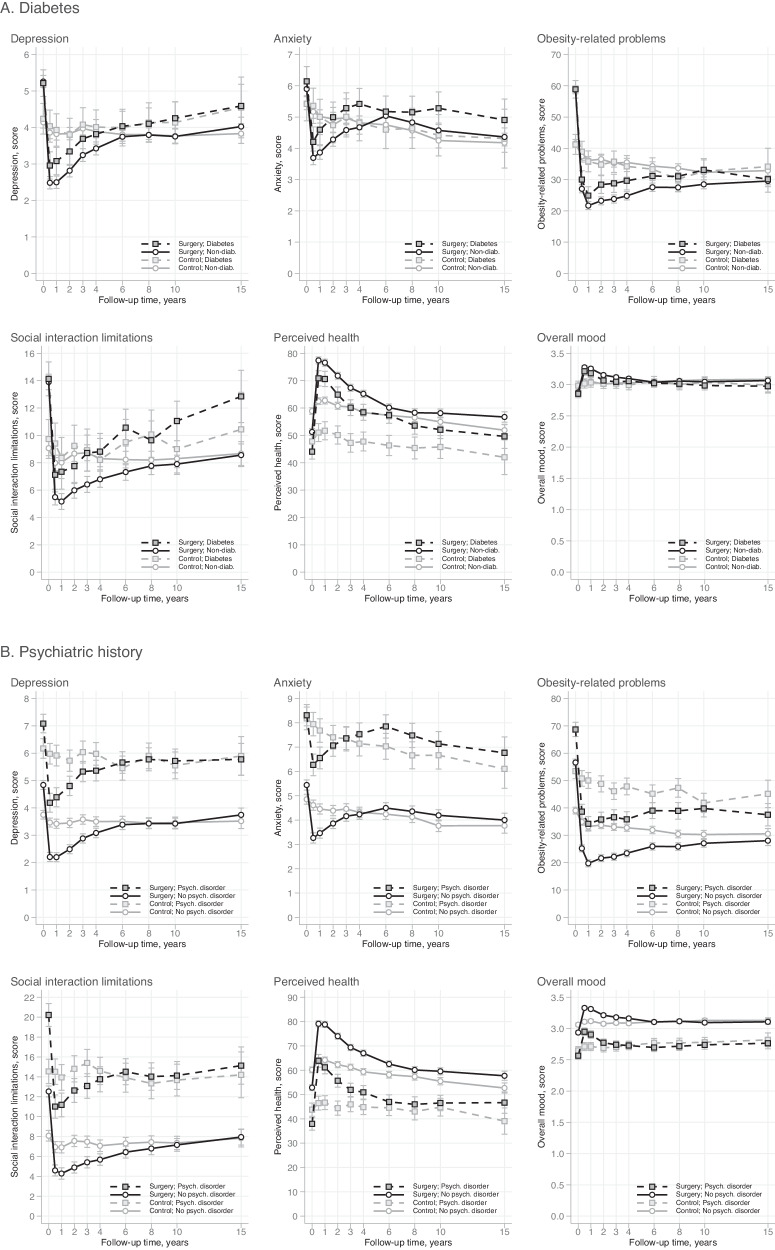
Absolute levels of HRQoL by baseline health status and the treatment group over 15 years of follow-up. **A** Mean scores and 95% CIs by diabetes status in the SOS surgery and control groups; **B** mean scores and 95% CIs by history of psychiatric disorder in the SOS surgery and control groups. Higher scores for perceived health (score range 0–100) and overall mood (score range 1–4) represent better HRQoL. Higher scores for anxiety and depression (score range 0–21) and obesity-related problems and social interaction limitations (score range 0–100) represent worse HRQoL.

Figures 2 and 3 display absolute levels of HRQoL scales by selected sociodemographic factors (age, sex) and baseline health status. Although pre-existing psychiatric disorder was not significantly associated with 15-year HRQoL changes in the surgery group (Table 3), surgical and control patients with a history of psychiatric disorder had worse perceived health and overall mood, and more depression, anxiety, obesity-related problems and social interaction limitations than those without such history from baseline to 15 years (Fig. 3). Additionally, in both treatment groups, women tended to report more obesity-related problems and younger participants (<47.8-year-olds) had higher anxiety levels than men and older participants, respectively, during the 15-year study period (Fig. 2).

### Comparison between 15-year study completers and non-completers

In the surgery and control groups, 923 and 758 patients participated in the 15-year follow-up, respectively. Non-participants had worse overall mood (*p* = 0.005 and *p* < 0.001 for surgical and control patients), and more depression (*p* = 0.015 and *p* = 0.016), obesity-related problems (*p* = 0.005 and *p* = 0.012) and social interaction limitations (*p* = 0.009 and *p* < 0.001) at baseline than participants (Supplementary Table 3). Poorer perceived health (*p* < 0.001 for surgical patients), higher BMI (*p* = 0.001 for controls) and lower proportion of men (*p* = 0.007 for controls) were also observed in non-participants. All these baseline differences were trivial, or in one case small, in effect size, however.

## Discussion

The present study contributes to the still limited knowledge on the long-term (i.e., ≥10 years) HRQoL development following bariatric surgery and its pre-operative predictors. Compared to control patients, surgical patients experienced greater 15-year improvements in mental, physical, social, and obesity-related domains of HRQoL (after adjustment for baseline differences between the groups). The difference in the long-term change was large for obesity-related problems, but otherwise the differences were small or trivial. While HRQoL was initially worse in the surgery than in the control group due to self-selection of surgical treatment, surgical patients had better physical and obesity-related HRQoL at the 15-year follow-up. In the surgery group, patients with pre-existing diabetes experienced smaller reductions in social interaction limitations and depression than those without diabetes. Albeit those with a history of psychiatric disorder reported lower HRQoL in all domains of functioning than those without such history over the 15-year study period, there was no clear evidence for differences in the long-term improvements between the two groups.

Our results regarding the 15-year changes in HRQoL were mostly similar to those reported earlier by Karlsson et al. on a subset of SOS participants over 10 years of follow-up [20]. In line with that study [20] and a study by Kolotkin et al. [13], obesity-related HRQoL showed the greatest within-group (baseline vs. 15 years in the surgery group) and between-group (surgery vs. usual care) improvement after 15 years of follow-up. Obesity-related HRQoL denotes how bothered the respondent is by their obesity in a broad range of social activities (e.g., private gatherings at home, going to restaurants, trying on and buying clothes, intimate relations), and the observed improvement in the surgery group is probably a reflection of the 15% reduction in BMI at the 15-year follow-up. Although the respective change in BMI was minor in patients receiving usual care, they still reported a small, long-term reduction in obesity-related problems, potentially reflecting the effects of aging and accumulated life-experiences on how these social situations are perceived. In both treatment groups, women experienced more obesity-related problems than men over the entire study period. This observation could be related to the higher BMI inclusion criteria for women (BMI ≥ 38 kg/m^2^) compared to men (BMI ≥ 34 kg/m^2^) in this study, or to women’s higher likelihood to encounter weight-based stigma and discrimination [4, 31].

Despite the improvement in perceived health not being as notable as the reduction in obesity-related problems in surgical patients, it is noteworthy that the direction of change was opposite in the two treatment groups. While perceived health improved in the surgery group, it deteriorated in the control group over 15 years. These differences are likely to reflect the well-documented effects of bariatric surgery on the prevention and resolution of obesity-related co-morbidities [7–10].

Mental health status demonstrated small improvements 15 years after bariatric surgery as indicated by the scales measuring anxiety, depression, and overall mood. Whereas depression and mood improved more following surgical than non-surgical treatment (between-group differences being small in size), the alleviation of anxiety symptoms was roughly similar in both treatment groups in accordance with earlier findings [20]. Epidemiological evidence implies that although anxiety disorders are chronic, there is a natural decrease in the prevalence rates with older age [32]. This was also evident in the present study as older age was related to lower and decreasing levels of anxiety over the study period in the surgery and control groups. It is thus possible that aging has influenced the course of anxiety symptoms in both treatment groups. The surgery group also showed a small long-term reduction in health-related limitations in social life, but in the control group, the corresponding change was negligible.

An important aspect to consider regarding the effect of surgical vs. non-surgical obesity treatment on subsequent HRQoL outcomes is the fact that the SOS participants who initially had worse HRQoL were more likely to select (as a co-decision with the physician) bariatric surgery than non-surgical treatment. This has at least two possible consequences. On the one hand, surgical patients possessed a higher likelihood of HRQoL improvement than control patients, while on the other hand, HRQoL had to improve in surgical patients to reach a HRQoL level similar to control patients. Although we controlled for these baseline differences in the analyses, it is still possible that they influence the observed effects of surgery (vs. usual care) on HRQoL development.

We further extended Karlsson et al.’s study [20] by examining whether long-term HRQoL changes in the surgery group varied according to sociodemographic characteristics, pre-operative health status or surgery type. Pre-existing diabetes emerged as one pertinent factor: surgical patients with baseline diabetes experienced smaller improvements in depression and health-related limitations in social life than those without diabetes. In contrast, the 5-year prospective study of 7000 gastric bypass patients from the Scandinavian Obesity Surgery Register noted that physical, social and obesity-related HRQoL (as measured by the RAND Short Form-36 and the OP scale) improved slightly more in patients with pre-operative diabetes [19]. While these conflicting results might be partly attributable to differences in the primary surgery type (100% vs. 13% of patients underwent gastric bypass) and follow-up time (5 vs. 15 years) between the two studies, the impact of pre-operative diabetes on long-term HRQoL outcomes clearly requires further research.

Additionally, we observed that obesity-related problems decreased more following gastric bypass than other surgery types in line with the fact that this surgical method is associated with the largest long-term weight loss [10]. It is, however, noteworthy that the present analyses did not reveal other significant differences between the three surgery types in terms of long-term changes in mental, physical and social domains of functioning. A recent systematic review on 5-year post-operative psychological outcomes also concluded that the most common surgery types (gastric bypass, sleeve gastrectomy, laparoscopic adjustable gastric banding) did not have significantly different effects on depression and anxiety outcomes [33].

While patients treated medically for depression prior to gastric bypass experienced less improvement in HRQoL than patients without such treatment in the Scandinavian Obesity Surgery Register [19], we did not detect significant differences in 15-year HRQoL changes between surgical patients with and without pre-existing psychiatric disorder. Thus, in our study, having a history of psychiatric disorder did not appear to impede long-term HRQoL improvements after bariatric surgery. Nonetheless, those with such history reported considerably poorer mental, physical, social and obesity-related HRQoL over the whole study period. Our previous findings from the SOS study also highlight the relevance of pre-existing psychiatric conditions in surgical patients, since pre-operative health care visits for self-harm or mental disorders and psychiatric drug use predicted higher risk of subsequent non-fatal self-harm and suicide [34].

American Society for Metabolic and Bariatric Surgery recently issued a position statement on pre-operative health optimization prior to metabolic and bariatric surgery. The position statement highlights that for patients with pre-existing psychiatric conditions, it is essential that their symptoms are adequately managed and that a behavioral health provider monitors and supports the patient after surgery [35]. Our present observations regarding different domains of HRQoL together with previous evidence [11] imply that regular psychosocial monitoring and support is important after bariatric surgery (for all patients, but particularly for those with prior psychiatric problems) due to possible setbacks in positive HRQoL development after the first or second post-operative year.

The main strengths of our study include the assessment of key HRQoL dimensions ten times over 15 years of follow-up in a relatively large sample of surgical and control patients, as well as the use of diverse information on the pre-operative situation to identify potential risk groups for long-term adverse HRQoL outcomes. Both within-group (baseline vs. 15 years) and between-group (surgery vs. usual care) changes were examined in this matched, non-randomized study to provide a detailed assessment of HRQoL development. Future research is, however, required to examine how post-operative changes in health status associate with long-term HRQoL changes to further inform clinical practice. Certain limitations of the present study should also be considered. Due to the non-randomized study design, we cannot eliminate the possibility that the choice of bariatric surgery or usual care affect the observed differences in HRQoL development between the two treatment groups. As is common in the long-term prospective studies with regular follow-up visits, there was non-participation over time. Surgical and control patients who did not attend 15-year follow-up tended to have lower HRQoL at baseline than those who attended, but these differences were mostly trivial in magnitude. Moreover, it should be noted that this pattern of non-participation was similar in the surgery and control groups.

## Conclusions

Obesity-related HRQoL showed a large 15-year improvement after bariatric surgery and particularly after gastric bypass. Small, long-term improvements in physical, mental, and social HRQoL were also noted. Patients receiving usual care reported small (obesity-related problems, anxiety) or trivial (depression, overall mood) long-term improvements in HRQoL, while their perceived health slightly deteriorated. Obesity-related HRQoL was the only domain showing a large difference in 15-year changes between the treatment groups, which is likely to reflect the considerable weight reduction associated with bariatric surgery. Surgical patients with pre-existing diabetes or psychiatric disorder had a higher likelihood of worse HRQoL outcomes than those without such conditions, thereby representing groups of patients for whom more intensive post-operative psychosocial monitoring and associated support might be vital.

## Supplementary information

Supplementary Tables and Figures

## Data Availability

The datasets analysed during the current study are not publicly available due to patient agreements and the sensitive nature of the patient data. The data are subject to legal restrictions according to national legislation. Confidentiality regarding personal information in studies is regulated in the Public Access to Information and Secrecy Act (SFS 2009:400), OSL. There is a possibility to apply to get access to public documents that an authority holds. In this case, the University of Gothenburg is the specific authority that holds the documents. A request to get access to public documents can be rejected or granted with reservations. If the authority refuses to disclose the documents the applicant is entitled to get a written decision that can be appealed to the administrative court of appeal.

## Code availability

All statistical analyses were performed with Stata software, version 15.1 (StataCorp). Relevant code are available from the corresponding author on request.
