# Supplementary material for: Fifteen-year changes in health-related quality of life after bariatric surgery and non-surgical obesity treatment
Source: Int J Obes (Lond). 2024 Jun 20;48(10):1447–56. doi: 10.1038/s41366-024-01572-w (PMC11420074; doi:10.1038/s41366-024-01572-w)
Supplement: Supplementary file 1 — Supplementary Tables and Figures [file 41366_2024_1572_MOESM1_ESM.pdf]

Supplementary Table 1. International Classification of Diseases (ICD) codes used to identify patients with health-care visits for mental disorders, substance abuse and non-fatal self-harm.

Condition	ICD-8	ICD-9	ICD-10
Mental disorders	290-315	290-319	Chapter F
Substance abuse	291; 394,3; 303-304; 970,00; 971; 989,99	291-292, 303-304, 305A, 305B, 305X, 357F, 425F, 535D, 571A, 571B, 571C, 571D	F10-F19, G621, I426, G721, K292, K700-K704, K709, K852, K860, G312, Z714-Z715, Z721-Z722
Non-fatal self-harm	E950-959, E980-989	E950-959, E980-989	X60-84, Y10-34, Y870

Supplementary Table 2. Fifteen-year mean changes in HRQoL scales by baseline education and relationship status and surgery type in the SOS surgery group estimated using mixed-models.[1]

	Education		Relationship status		Surgery type		
	Basic N=1393	Higher N=614	Not married N=547	Married/partner N=1460	Banding N=376	VBG N=1365	GBP N=266
Perceived health[2]							
Mean	4.7	5.3	3.7	5.4	3.9	5.6	6.6
95% CI	2.5 to 7.0	1.9 to 8.6	-0.1 to 7.4	3.3 to 7.5	-0.5 to 8.3	3.4 to 7.8	1.4 to 11.7
P[4]	0.799		0.429		ref.	0.451	0.412
Anxiety[3]							
Mean	-1.4	-1.2	-1.4	-1.3	-1.9	-1.3	-1.4
95% CI	-1.8 to -1.1	-1.7 to -0.7	-2.0 to -0.9	-1.7 to -1.0	-2.5 to -1.3	-1.6 to -1.0	-2.1 to -0.7
P[4]	0.455		0.801		ref.	0.100	0.344
Depression[3]							
Mean	-1.1	-1.0	-0.9	-1.1	-1.4	-1.0	-1.0
95% CI	-1.4 to -0.8	-1.4 to -0.5	-1.4 to -0.4	-1.4 to -0.8	-2.0 to -0.8	-1.3 to -0.8	-1.7 to -0.4
P[4]	0.619		0.461		ref.	0.288	0.475
Overall mood[2]							
Mean	0.2	0.2	0.2	0.2	0.2	0.2	0.2
95% CI	0.1 to 0.2	0.1 to 0.2	0.1 to 0.2	0.1 to 0.2	0.1 to 0.3	0.1 to 0.2	0.1 to 0.3
P[4]	0.832		0.695		ref.	0.378	0.854
Obesity-related problems[3]							
Mean	-27.6	-30.7	-27.3	-29.1	-27.1	-28.2	-35.3
95% CI	-29.6 to -25.5	-33.8 to -27.6	-30.7 to -23.9	-31.0 to -27.1	-31.1 to -23.1	-30.2 to -26.2	-40.0 to -30.6
P[4]	0.094		0.369		ref.	0.525	**0.009**
Social interaction limitations[3]							
Mean	-4.4	-4.4	-3.5	-4.7	-5.2	-4.3	-6.1
95% CI	-5.3 to -3.5	-5.8 to -2.9	-5.0 to -1.9	-5.7 to -3.8	-7.1 to -3.4	-5.2 to -3.3	-8.3 to -4.0
P[4]	0.967		0.168		ref.	0.446	0.481

[1]Analyses adjusted for sex, age, baseline BMI and baseline HRQoL scale.

[2]Increased scores represent improved perceived health and overall mood, respectively.

[3]Decreased scores represent reduced anxiety, depression, obesity-related problems and social interaction limitations, respectively.

[4]Difference in 15-year change between the groups (adjusted for sex, age, baseline BMI and the respective HRQoL scale at baseline).

Supplementary Table 3. Baseline characteristics of 15-year study completers and non-completers (excluding deaths before 15-year follow-up) in the SOS control and surgery groups.

	Control				Surgery			
	Completers N=758	Non-completers N=1062-1065	P value[1]	Effect size[2]	Completers N=923	Non-completers N=920-922	P value[1]	Effect size[2]
Age (years)	48.2 (6.0)[3]	48.5 (6.4)	0.323	0.05	47.2 (5.8)	46.8 (6.0)	0.101	0.08
Men (%)	31.0	25.2	0.007		30.1	26.0	0.055	
BMI (kg/m^2)	39.7 (4.5)	40.4 (4.7)	0.001	0.15	42.2 (4.4)	42.5 (4.5)	0.104	0.08
Perceived health[4]	59.0 (25.6)	57.2 (25.6)	0.134	0.07	52.7 (24.1)	48.6 (25.1)	<0.001	0.17
Anxiety[4]	5.2 (4.5)	5.5 (4.6)	0.177	0.06	5.8 (4.4)	6.0 (4.5)	0.455	0.03
Depression[4]	3.9 (3.2)	4.3 (3.7)	0.016	0.12	5.0 (3.6)	5.4 (3.6)	0.015	0.11
Overall mood[4]	3.1 (0.6)	3.0 (0.6)	<0.001	0.16	2.9 (0.6)	2.8 (0.6)	0.005	0.13
Obesity-related problems[4]	39.4 (26.9)	42.6 (26.8)	0.012	0.12	56.7 (26.5)	60.2 (26.9)	0.005	0.13
Social interaction limitations[4]	7.6 (10.6)	10.0 (13.0)	<0.001	0.20	12.8 (12.6)	14.4 (14.0)	0.009	0.12

[1]Differences between the completers and non-completers were tested using t-tests (continuous variables) or Fisher's exact tests (categorical variables).

[2]Effect size (between-group) was calculated by dividing the difference in baseline mean between the completers and non-completers by the pooled standard deviation of mean. It was judged against the following criteria: trivial (0 to <0.2), small (0.2 to <0.5), moderate (0.5 to <0.8) and large (≥0.8).(30)

[3]Mean (SD), all such values.

[4]Perceived health: higher scores represent better perceived health (score range 0–100). Anxiety and depression: higher scores represent more symptoms (score range 0–21). Overall mood: higher scores represent more positive mood states (score range 1–4). Obesity-related problems and social interaction limitations: higher scores represent more dysfunction (score range 0–100).

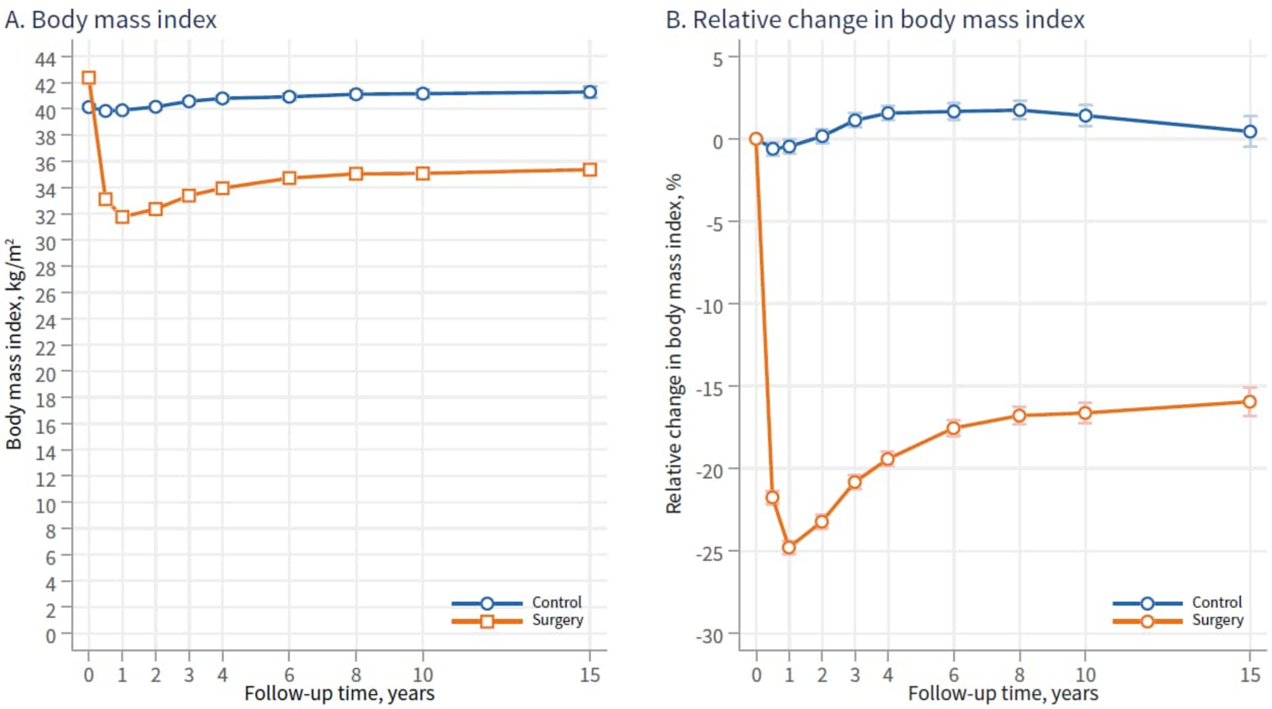

Supplementary Figure 1. Absolute levels (mean and 95% CI) of body mass index (A) and relative changes (mean and 95% CI) in body mass index (B) by treatment group over 15 years of follow-up.
